# Gliclazide Prevents 5-FU-Induced Oral Mucositis by Reducing Oxidative Stress, Inflammation, and P-Selectin Adhesion Molecules

**DOI:** 10.3389/fphys.2019.00327

**Published:** 2019-03-26

**Authors:** Cristiane Assunção da Costa Cunha Mafra, Roseane Carvalho Vasconcelos, Caroline Addison Carvalho Xavier de Medeiros, Renata Ferreira de Carvalho Leitão, Gerly Anne de Castro Brito, Deiziane Viana da Silva Costa, Gerlane Coelho Bernardo Guerra, Raimundo Fernandes de Araújo, Aldo Cunha Medeiros, Aurigena Antunes de Araújo

**Affiliations:** ^1^Postgraduate Programs in Oral Science, Department of Biophysics and Pharmacology, Federal University of Rio Grande Norte, Natal, Brazil; ^2^Postgraduate Program in Public Health, Department of Biophysics and Pharmacology, UFRN, Natal, Brazil; ^3^Department of Biophysics and Pharmacology, UFRN, Natal, Brazil; ^4^Postgraduate Program in Biological Science and Rede Nordeste de Biotecnologia/Renorbio, Federal University of Rio Grande Norte, Natal, Brazil; ^5^Postgraduate Programs in Pharmacology and Morphology, Department of Morphology/Pharmacology, Federal University of Ceará, Fortaleza, Brazil; ^6^Postgraduate Programs in Postgraduate Program in Biological Science/Pharmaceutical Science, Department of Biophysical and Pharmacology, UFRN, Natal, Brazil; ^7^Postgraduate Programs in Functional and Structural Biology and Health Science, Department of Morphology, UFRN, Natal, Brazil; ^8^Postgraduate Programs in Health Science, Department of Surgery, UFRN, Natal, Brazil; ^9^Postgraduate Oral Science, Postgraduate Programs in Pharmaceutical Science, Department of Biophysics and Pharmacology, Federal University of Rio Grande Norte, Natal, Brazil

**Keywords:** 5-fluorouracil, oral mucositis, gliclazide, oxidative stress, inflammation

## Abstract

Oral mucositis (OM) is one of the main side effects of the head and neck cancer treatment, particularly radiotherapy and/or chemotherapy. OM is characterized by ulcers, erythema, dysphagia, xerostomia, and increased susceptibility to opportunistic infections. In the perspective of finding pharmacological therapies to prevent inflammation and ulceration of OM, the investigation of the pleiotropic effect of commercial drugs is needed, among them gliclazide, an antidiabetic drug. This study aimed to evaluate the effect of gliclazide in an experimental OM model induced by 5-fluorouracil. Male hamsters were pre-treated with oral gliclazide (1, 5, or 10 mg/kg) for 10 days. Cheek pouch samples were subjected to histopathological and immunohistochemical analysis (COX_2_, iNOS, MMP-2, NFκB P65, GPx) and imunofluorescence (P-selectin). IL-1β and TNF-α levels, Myeloperoxidase activity (MPO) and malondialdehyde (MDA) levels were investigated by ultraviolet-visible spectroscopy analysis. NFκB NLS P50 protein levels were analyzed by western blotting. The group treated with gliclazide at a dose of 10 mg/kg showed presence of erythema, no evidence of erosion, and absence of mucosal ulceration with a score of 1 (1–2) (*p* < 0.01). Histopathological data for the group treated with gliclazide 10 mg/kg showed re-epithelialization, discrete mononuclear inflammatory infiltrate and absence of hemorrhage, edema, ulcers and abscesses with a score of 1 (1–1) (*p* < 0.01). Treatment with gliclazide 10 mg/kg reduced MPO activity (*p* < 0.001), MDA levels (*p* < 0.001) and NFκB NLS P50 (*p* < 0.05) protein levels, resulting in low immunostaining to Cox-2, iNOS (*p* < 0.05), NFκB P65 (*p* < 0.05), and negative immunoreaction to MMP-2 (*p* < 0.001). However, it appeared that for Gpx1, the staining was restored in the GLI 10-FUT group compared with 5FUT/saline (*p* < 0.05). Immunofluorescence revealed decreased levels of P-selectin (*p* < 0.001) after treatment with gliclazide 10 mg/kg (*p* < 0.05). In summary, gliclazide accelerated mucosal recovery and reduced oxidative stress and inflammation in the 5-FU-induced OM in hamsters.

## Introduction

Oral mucositis (OM) is one of the main toxicities of cancer therapy, affecting up to 60–100% of patients undergoing radiotherapy and/or chemotherapy (Chaveli-Lopez, 2014). The term “mucositis” refers to an inflammatory process that affects parapharyngeal, esophageal and gastrointestinal regions, characterized by acute inflammation in the oral mucosa, and clinically manifested by painful ulcers, erythema, dysphagia, xerostomia and increased susceptibility to opportunistic infections with the risk of sepsis in neutropenic patients (Jeddi et al., 2010; Mauz-Korholz et al., 2017; Oronsky et al., 2018). Therapeutic regimen and antineoplastic agent contribute to the severity of the lesions (Lopez-Castano et al., 2005).

Among the chemotherapeutic agents, methotrexate, irinotecan and 5 Fluorouracil (5-FU) are associated with high prevalence of oral and intestinal mucositis, as well as anorexia, nausea and diarrhea (Sonis et al., 2004; Stein et al., 2010). 5-FU particularly compromises the DNA and RNA biosynthesis due to the inhibition of the thymidylate synthase (TS) enzyme by its active metabolite, 5-fluoro-2′-deoxyuridine 5′-monophosphate (FdUMP). The 5-FU chemotherapy involves a series of metabolization stages to exert its antitumor action, in which some metabolites can be incorporated into DNA and RNA molecules, or in the case of FdUMP, inhibit enzymes involved in processing these molecules, compromising the cell growth (Matuo et al., 2009). In virtue of its high mitotic activity, the oral mucosa epithelium suffers greater damage by antineoplastic agents that become more effective in rapidly proliferating cells such as bone marrow, hair follicles and epithelial cells of the digestive tract (Tamaki et al., 2003).

The initiation phase of mucositis is characterized by radio or chemotherapy-induced direct DNA damage, that results in injury to the basal epithelial, submucosal, and endothelial cells. These cells release endogenous damage-associated molecular patterns (DAMPs), that further damage cell membranes, stimulate macrophages, and trigger molecules that activate transcription factors, including nuclear factor (NF)κB (Shankar et al., 2017). The NFκB pathway has long been considered a prototypical proinflammatory signaling pathway, with expression of proinflammatory genes including cytokines, chemokines, and adhesion molecules (Lawrence, 2009).

Treatment of OM symptoms includes anti-thermal, anti-inflammatory, antimicrobial, cryotherapy, and antiseptic analgesics, but there are still a few prophylactic therapies, namely laser therapy and keratinocyte growth factor intravenous administration (Viet et al., 2014; Chaveli-Lopez and Bagan-Sebastian, 2016).

In the perspective of finding pharmacological therapies to prevent inflammation and ulceration of OM, we highlight the need to investigate the pleiotropic effect of commercial drugs, specifically among them gliclazide, an antidiabetic drug classified as second generation sulfonylurea, with secondary anti-oxidant and anti-inflammatory effects (Drzewoski and Zurawska-Klis, 2006; Sliwinska et al., 2012). Gliclazide has an inhibitory effect on adhesion molecules, independent of its hypoglycemic effect (Papanas et al., 2006). The increase in cell adhesion molecule (CAM) expression following treatment with irinotecan can directly affect the normal inflammatory response required after an injury, and can also impact on cell kinetics through indirect effects on the subepithelial extracellular matrix (ECM) in mucositis (Al-Dasooqi et al., 2017). Thus, the present study aims to investigate the effect of gliclazide on an experimental OM model induced by 5-FU in hamsters.

## Materials and Methods

### Animals

Thirty (30) male Golden Sirian hamsters with a mean weight of 140–180 g were used. The hamsters were provided by the breeding room in the Department of Biophysics and Pharmacology under the appropriate hygiene conditions, at a temperature of 22°C and light/dark cycle 12 h. The animals were housed in individual cages and had free *ad libitum* access to water and feed. Experimental protocols were performed according to the norms and guidelines approved by the Federal University of Rio Grande Norte Committee for the Ethical treatment of research animals (protocol No. 031/2016).

### Experimental Groups and Design

The experimental model was performed according to the methodology described by Sonis et al. (1990). Hamsters were randomly divided into six experimental groups (05 animal/group): (1) Saline group received intraperitoneal (i.p.) 0.9% saline solution on the 1st and 2nd experimental days to mimic 5-FU injection, saline by gavage for 10 days; (2) mechanical trauma (MT) group received i.p. saline administration on the 1st and 2nd experimental days; then the animals were submitted to MT in the right cheek mucosa on the 4th day and received saline by gavage for 10 days; (3) 5-FUT/saline group received 5-FU injections on the 1st and 2nd days (60 and 40 mg/kg i.p., respectively), then it was submitted to MT in the right cheek mucosa on the 4th day and saline by gavage for 10 days; (4) GLI 1-FUT; (5) GLI 5-FUT; and (6) GLI 10-FUT received gliclazide by gavage for 10 days, 5-FU injections on the 1st and 2nd days (60 and 40 mg/kg i.p.) and was submitted to MT in the right cheek mucosa on the 4th day. At day 10, hamsters were euthanized by i.p. injection of 2% thiopental (100 mg/kg) and blood and tissue samples were collected for subsequent analyses.

### Macroscopic Analysis

After euthanasia of the animals, their oral mucosa were photographed for macroscopic analysis. The evaluated parameters were the presence and intensity of erythema, hyperemia, hemorrhage, ulcers and abscesses, classified according to the following scores (Medeiros et al., 2011):

• Score 0: completely healthy mucosa. No erosion or vasodilation.
• Score 1: presence of erythema, but no evidence of mucosal erosion.
• Score 2: severe erythema, vasodilatation and superficial erosion.
• Score 3: ulcer formation on one or more faces, but not affecting more than 25% of the surface area of the pouch. Severe erythema and vasodilation.
• Score 4: cumulative ulcer formation of about 50% of the pouch surface area.
• Score 5: virtually complete ulceration of the mucosa of the pouch. Impossibility of mucosal exposure.

### Histopathological Analysis

The oral mucosa was fixed in 10% buffered formaldehyde solution for 24 h, then dehydrated and embedded in paraffin. Each sample was sectioned in 4 μm thick serial sections followed by hematoxylin-eosin (H&E) staining. The specimens were analyzed by light microscopy of a simple-blind form for the inflammatory aspects, such as presence and intensity of the cellular infiltration, dilation and vascular engorgement, hemorrhage, edema, ulcers, and morphological characteristics suggestive of abscesses, classified according to the standardized scores (de Araujo et al., 2015) with adaptations, as follows:

• Score 0: epithelium and connective tissue without vasodilation; absent or discrete cell infiltrate; absence of bleeding, edema, ulcers and abscesses.
• Score 1: discrete vascular engorgement; areas of reepithelialization; discrete cell infiltrate, with greater number of mononuclear leukocytes, absence of hemorrhage, edema, ulcers and abscesses.
• Score 2: moderate vascular engorgement; epithelial hydropic degeneration (vacuolization); moderate cellular infiltrate, with a predominance of mixed leukocytes; presence of hemorrhagic areas, edema, and occasional small ulcers; absence of abscesses.
• Score 3: marked vascular engorgement; marked vasodilation; accentuated, mixed cell infiltrate; presence of hemorrhagic areas, edema, abscesses, and extensive ulcers.

### Immunohistochemistry

Sections of 3-μm thick tissue embedded in paraffin were arranged on 3-aminopropyltriethoxysilane slides (Sigma Chemical Co., St. Louis, MO, United States) for immunohistochemical analysis. The samples were subsequently dewaxed with xylol, rehydrated in alcohols and washed in water and buffer solution. Antigen retrieval was performed using citrate and then blocking with bovine serum albumin (BSA) was performed at room temperature for 2 h. Endogenous peroxidase blockade was performed using 3% hydrogen peroxide for 10 min. Next, the slides were incubated for 18 h in a humidified chamber at 4°C with the following primary polyclonal antibodies (Santa Cruz Biotechnology, Interprise, Brazil): anti-COX-2 1:400; anti- metalloproteinase-2 (MMP-2) 1:400; anti-iNOS 1:400; anti-GPx 1:400, anti-NFκB P65 1:400. The sections were treated after washing with PBS.

Analyzed samples were considered positive for the expression of COX-2, MMP-2, iNOS, GPx, and NFκB P65 in mucosal tissues that exhibited brown cytoplasmic or membrane staining. A semi-quantitative analysis was performed for all antibodies following the methodology proposed by Curra et al. (2013), with adaptations for this study. All representative areas of the lesion were blindly examined under a light microscope (Olympus CH30, Olympus Japan Co., Tokyo, Japan) under 400× magnification. Specimens were classified according to the immunolabeled area with the following scores: 0 (absence of immunolabeling), 1 (<10% cell positive/discrete immunolabeling), 2 (11–50% cell positive/moderate immunolabeling), and 3 (>50% positive cells/intense immunolabeling).

### Confocal Immunofluorescence

The same immunohistochemistry protocol was used for confocal immunofluorescence, but the tissue sections were incubated overnight at 4°C with the primary antibody (Santa Cruz Biotechnology, Santa Cruz, CA, United States) to P-selectin 1:200 for 1 h. After washing in phosphate buffered saline/0.2% Triton X-100 for 5 min, the tissues were then incubated with Alexa Fluor 488-conjugated goat anti-rabbit (Abcam Inc., Cambridge, MA, United States) secondary antibody (1:500) for 1 h. Fluorescent images were obtained on a Carl Zeiss laser scanning microscope (LSM 710, 20 × objective, Carl Zeiss, Jena, Germany). Tissue reactivity in all groups was assessed by computerized densitometry analysis of the digital images captured with the confocal immunofluorescence microscope. Average densitometric values were calculated in Image J software (Wayne Rasband; National Institutes of Health, Bethesda, MD, United States).

### Quantification of Pro-inflammatory Cytokines (TNF-α, IL-1β) by Elisa Assay

The oral mucosa of animals treated with gliclazide (1, 5, 10 mg), 5-FU and saline were collected in 2 ml eppendorfs and conditioned under freezing at -80°C for subsequent cytokine dosing (TNF-α and IL-1β) following the protocol of Safieh-Garabedian et al. (1995). TNF-α and IL-1β concentrations were measured using the enzyme-linked immunosorbent assay technique (ELISA) using the Duoset kit (R&D systems). First, 96-well Elisa plates were incubated for 18 h at 4°C with 100 μl capture antibody for TNF-α and IL-1β. Three plate washes were subsequently performed with 300uL of tween20 wash buffer. Then, blocking was performed with 100 μl of 1% bovine albumin (BSA) in the wells. After blocking, samples (100 μl) and antibodies were added to obtain standard curves at various dilutions and incubated for 2 h at 4°C. Next, the plates were washed three times with 300uL wash buffer and incubated with detection antibodies for TNF-α and IL-1β for 2 h at 4ˆA°C. After the incubation period, the plates were washed again three times with wash buffer and incubated at room temperature for 20 min with 100 μl of diluted streptavidin at 1:200. Then the plates were washed with wash buffer and incubated at room temperature in the dark for 20 min with 100 μl of KIT Duo Set (R&D system). The enzyme reaction was quenched with 0.018 M H_2_SO_4_ solution and the absorbance was measured at 450 nm. The results were expressed in pg/ml.

### Malonaldehyde (MDA) Levels

Malonyldialdehyde (MDA) is an end product of lipid peroxidation. To quantify the increase in free radicals in gingival sample, MDA content was measured via the assay previously described (Bradley et al., 1982; Siddique et al., 2012). Gingival samples were suspended in Tris HCl 1:5 (w/v) buffer and minced with scissors for 15 s on an ice-cold plate. The resulting suspension was homogenized for 2 min with an automatic Potter homogenizer and centrifuged at 2500 × g at 4°C for 10 min. The supernatants were assayed to determine MDA content. The absorbance of each sample was measured at 586 nm. The results are expressed as nanomoles of MDA per gram of tissue.

### Myeloperoxidase (MPO) Assay

The extent of neutrophil accumulation in gingival tissue samples was measured by assaying MPO activity. Gingival tissue (4 samples per group) was harvested as described above and stored at -80°C until required for assay. After homogenization of tissue in Hexadecyltrimethylammonium bromide (1:20), 02 freeze/thaw cycles and centrifugation (5000 RPM for 20 min), the MPO activity in these samples (in units of MPO/mg tissue) was determined by a previously described colorimetric method 450 (Bradley et al., 1982; Souza et al., 2003).

### Immunoblotting

OM tissue samples were homogenized in RIPA lysis buffer (25 mmol/L Tris-HCL, pH 7.6; 150 mmol/L NaCl; 5 mmol/L EDTA; 1% NP40; 1% triton X-100; 1% sodium deoxycholate; 0.1% sodium dodecyl sulfatepolyacrylamide) and protease inhibitor (1 μl in 100 μl RIPA). After centrifugation (17 min, 4°C, 13,000 rpm), the supernatant was collected, and protein concentrations were determined by bicinchoninic acid assay (Thermo Fisher Scientific) according to the manufacturer’s protocol. Sodium dodecyl sulfatepolyacrylamide-polyacrylamide gel electrophoresis (10% or 8%) was performed with 40 μg of protein prepared in Laemmli sample buffer (BioRad) and denatured at 95°C for 5 min. The protein was transferred to a PVDF membrane (BioRad) for 2 h, blocked with 5% BSA for 1 h, incubated overnight with a primary antibody (mouse anti-β actin, sc-81178, 1:500, Santa Cruz Biotechnology; rabbit anti- NFKB NLS P50 -sc 114, 1:100, Santa Cruz Biotechnology and then incubated for 1 h and 30 min with a secondary antibody (goat anti-rabbit, 656120, 1:1000; or goat anti-mouse IgG, 626520, 1:500; Invitrogen). The bands were visualized with an ECL system applied according to the manufacturer’s instructions (BioRad). Chemiluminescence signal was detected with a ChemiDoc^TM^ XRS system (BioRad) and quantified densitometrically in the ImageJ software (NIH, Bethesda, MD, United States).

### Statistical Analysis

Data are presented as group means ± standard errors or as medians with ranges, as appropriate. Analysis of variance (ANOVA) followed by Bonferroni’s test was used to compare mean values across groups. The Kruskal–Wallis test followed by Dunn’s test was used to compare medians. The statistical analyses were conducted in Prism 5.0 software (GraphPad, La Jolla, CA, United States). A *p* < 0.05 indicated a statistically significant difference.

## Results

### Macroscopic and Histopathological Analyses

**FIGURE 1:**
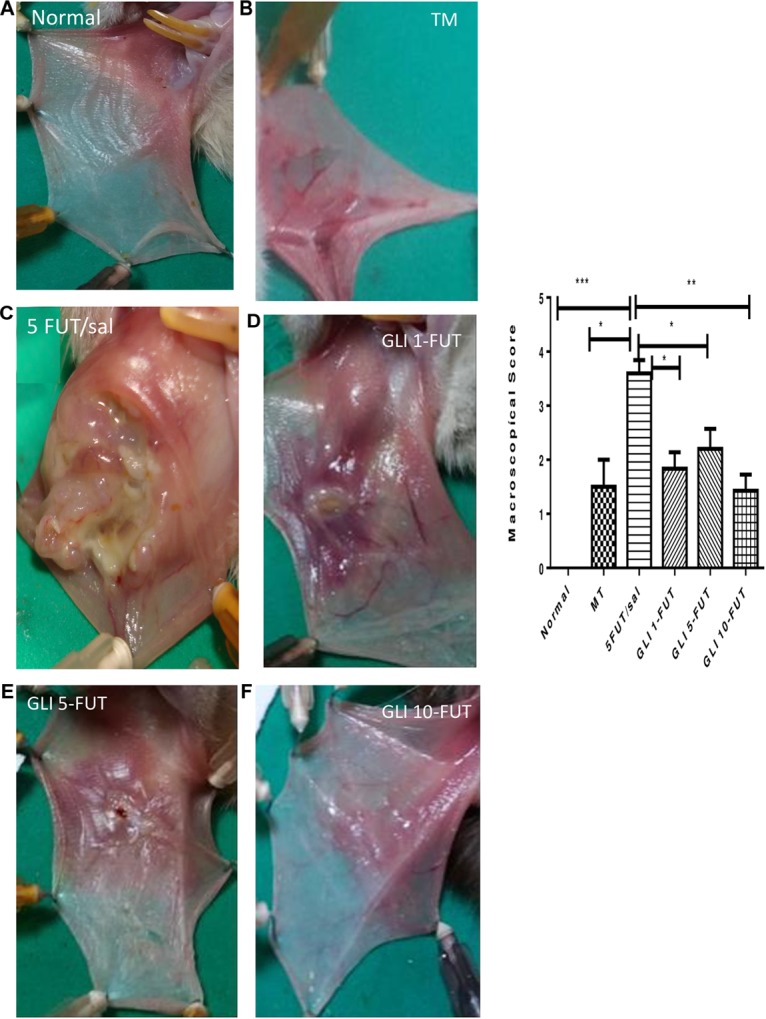
Macroscopic aspects of the samples. Normal hamster cheek pouch tissue **(A)**. Tissue subjected to mechanic trauma/MT **(B)**. 5-FUT/saline **(C)** showing erythema, hyperaemia, haemorrhagic areas, and extensive ulceration on the oral mucosa. Cheek pouch tissues treated with GLI 1-FUT **(D)**, GLI 5-FUT **(E)**, and GLI 10-FUT **(F)**. Graph demonstrates the comparison of scores of macroscopic features between groups (^∗^*p* < 0.05, ^∗∗^*p* < 0.01, ^∗∗∗^*p* < 0.001).

**FIGURE 2:**
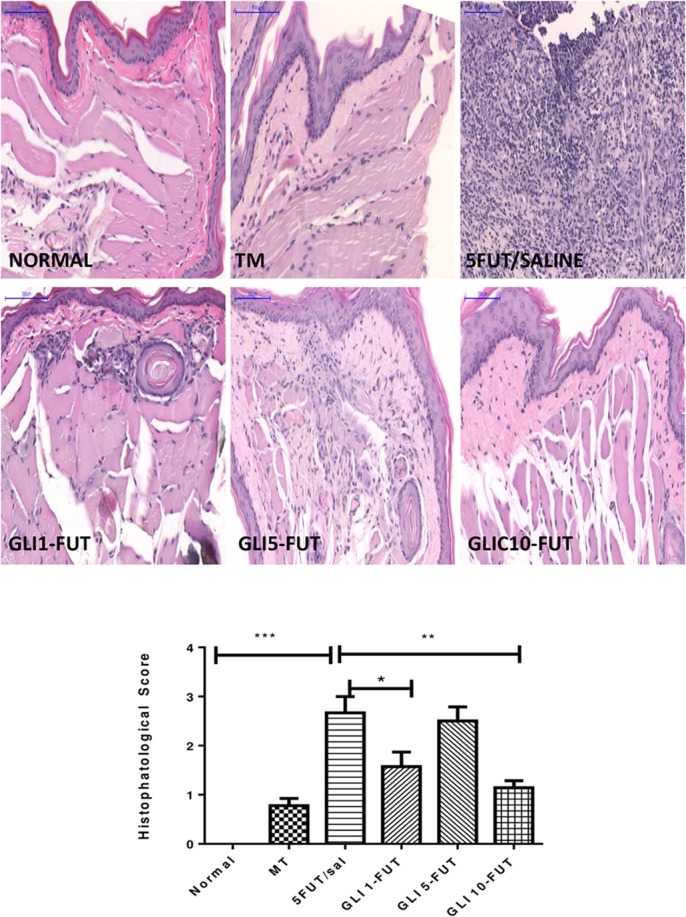
Photomicrograph showing the histopathological aspects of the mucosa of hamsters with 5-FU-induced oral mucositis treated with gliclazide. (SALINA): normal mucosa. (TRAUMA): discrete inflammatory infiltrate ^∗^. (5-FU): intense inflammatory infiltrate ^∗∗∗^; extensive ulceration with fibrinopurulent exudate ↔. (GLI-1FUT): focal areas of moderate inflammatory infiltrate ^∗∗^. (GLI-5FUT): focal areas with moderate inflammatory infiltrate ^∗∗^; engorged blood vessels.” (GLI-10FUT): Discrete inflammatory infiltrate ^∗^. (Scanner blades Panoramic Viewer – H&E, 200×). Histopathological scores of normal hamster cheek pouch tissue, tissue subjected to mechanic trauma/MT, 5FUT/saline, GLI 1-FUT, GLI 5-FUT, and GLI 10-FUT (^∗^*p* < 0.05, ^∗∗^*p* < 0.01, ^∗∗∗^*p* < 0.001).

The animals of the 5-FUT/saline group developed OM with clinical features such as severe erythema, extensive hyperemia, hemorrhagic areas, ulcers and abscesses, with a median of 4 (3–4), when compared to the normal group (*p* < 0.001; Figure 1). Histopathological characteristics in this group were vascular engorgement and marked vasodilatation, intense cellular infiltration (predominantly polymorphonuclear), presence of hemorrhagic areas, edema, abscesses and extensive ulcers, with score 3 (2–3), when compared to the normal group (*p* < 0.001; Figure 2). The best results for the macroscopic and histopathological analyzes were verified in the groups treated with gliclazide at a dose of 10 mg. Macroscopically, GLI 10-FUT revealed the presence of erythema, without evidence of erosion and ulcers with a score of 1 (1–2) (*p* < 0.01, Figure 1). These macroscopic results of GLI 10-FUT showed presence of discrete inflammatory process without damage to the oral mucosa. Histopathological data showed re-epithelialization, discrete mononuclear inflammatory infiltrate, and absence of hemorrhage, edema, ulcers and abscesses 1 (1–1) (*p* < 0.01, Figure 2).

### Oxidative Stress and Inflammation

**FIGURE 3:**
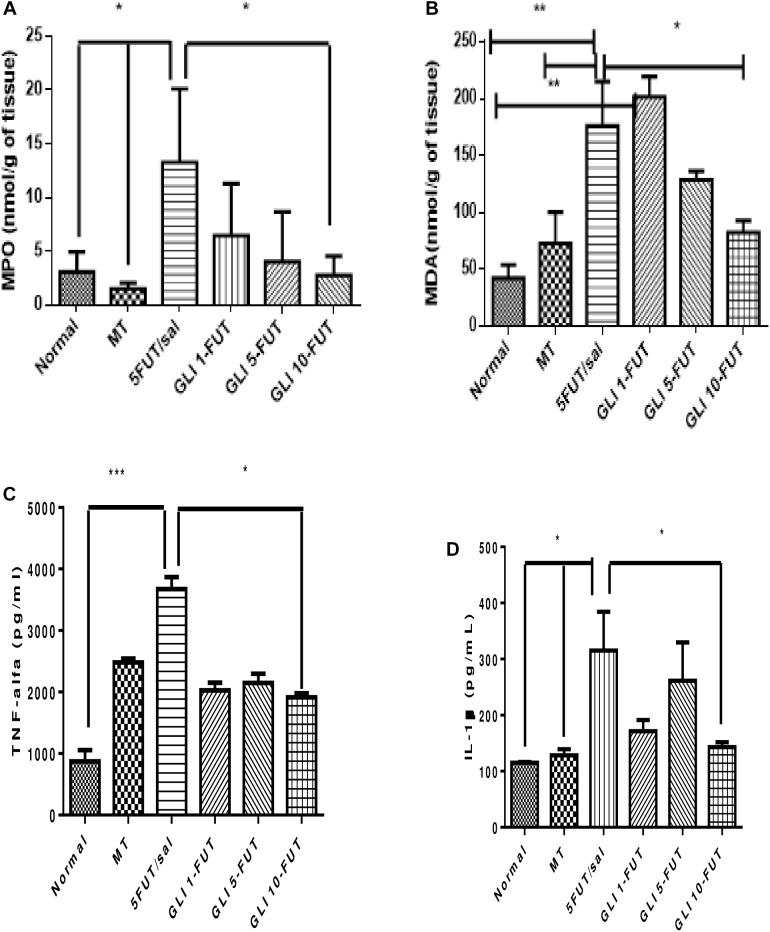
Levels of **(A)** MDA, **(B)** MPO activity, **(C)** IL-1 Beta, and **(D)** TNF-alfa in the normal hamster cheek pouch tissue, tissue subjected to mechanic trauma/MT, 5-FUT/saline, GLI 1-FUT, GLI 5-FUT, and GLI 10-FUT (^∗^*p* < 0.05, ^∗∗^*p* < 0.01, ^∗∗∗^*p* < 0.001).

MDA showed a significant increase in the 5-FUT/saline group (*p* < 0.01) and GLI 1-FUT (*p* < 0.05) compared to the normal group (*p* < 0.01). A significant reduction of MDA in the GLI 10-FUT group was observed when compared to the 5-FUT/saline (*p* < 0.05; Figure 3). Regarding the inflammatory response, there was a significant decrease in neutrophilic MPO activity in the GLI 10-FUT (*p* < 0.05).

**FIGURE 4:**
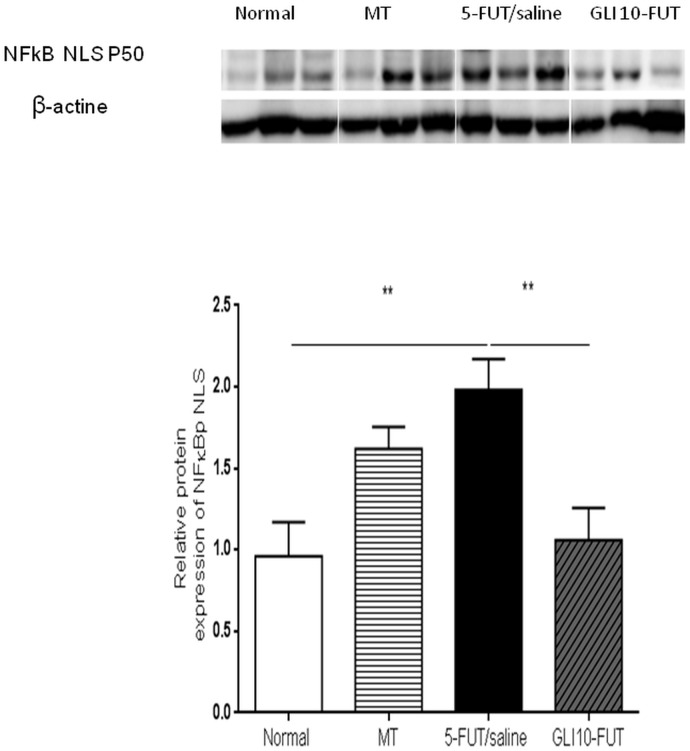
Representative images of NFκB P50 immunoblotting. The bands were visualized with an ECL system applied according to the manufacturer’s instructions (BioRad). The chemiluminescence signal was detected with a ChemiDoc^TM^ XRS system (BioRad) and densitometrically quantified in ImageJ software (NIH, Bethesda, MD). Normal hamster cheek pouch tissue, tissue subjected to mechanical trauma/MT, 5-FUT/saline, GLI 1-FUT, GLI 5-FUT, and GLI 10-FUT ^∗∗^*p* < 0.01).

Evaluation of inflammatory cytokines showed a significant reduction in TNF-α levels in the normal (*p* < 0.01) and GLI 10-FUT groups (*p* < 0.05) when compared to the 5-FUT/saline group. For IL-1β, a significant reduction was observed in the normal, MT and GLI 10-FUT groups compared to the 5-FUT/saline group (*p* < 0.05; Figure 3). Analysis of NFκB P50 protein expression levels in the normal and GLI 10-FUT groups were significantly lower than the 5-FUT/saline group (*p* < 0.05; Figure 4).

### Immunohistochemistry and Immunofluorescence Analyses

**FIGURE 5:**
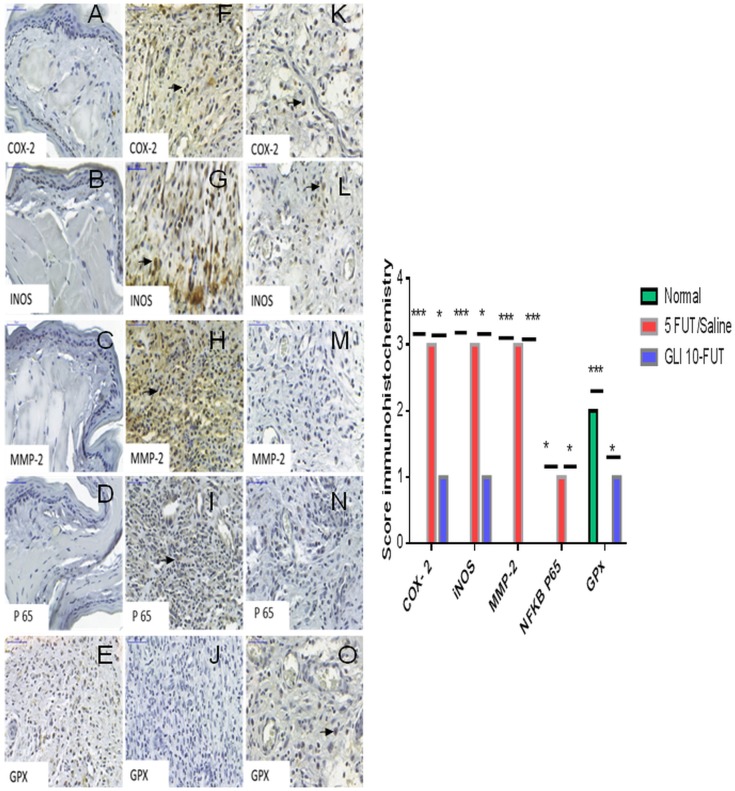
Immunostaining of COX-2, iNOS, MMP-2, NFkB P65, GPx, in oral mucositis. Absent reaction for COX-2 **(A)**, iNOS **(B)**, MMP-2 **(C)**, NFκB P65 **(D)**, in the normal group. Intense GPx **(E)** expression in the normal group. Intense COX-2 **(F)**, iNOS **(G),** and MMP-2 **(H)** expression in the 5-FUT/saline group. Mild immunoexpression of NFκB P65 **(I)** in 5-FUT/saline group. Absence of immunoreaction for GPx **(J)** in 5-FUT/saline group. Negative reaction for MMP-2 **(M)** and NFκB P65 **(N)** in GLI 10-FUT. Weak immunoexpression of COX-2 **(K)**, and iNOS **(L)**, demonstrating reduced expression of these proteins in the group treated with GLI 10-FUT. It appears that for GPx **(O)**, the coloration is being restored in the GLI 10-FUT group compared with 5 FUT/Saline group. Immunostaining in inflammatory cells (black arrow) (Panoramic Viewer, 400×). Graph shows immunohistochemical score for COX-2, iNOS, MMP-2, NFkB, GPx, in oral mucositis. Normal hamster cheek pouch tissue, tissue subjected to mechanical trauma/MT, 5-FUT/saline, GLI 1-FUT, GLI 5-FUT, and GLI 10-FUT (^∗^*p* < 0.05, ^∗∗^*p* < 0.01, ^∗∗∗^*p* < 0.001).

**FIGURE 6:**
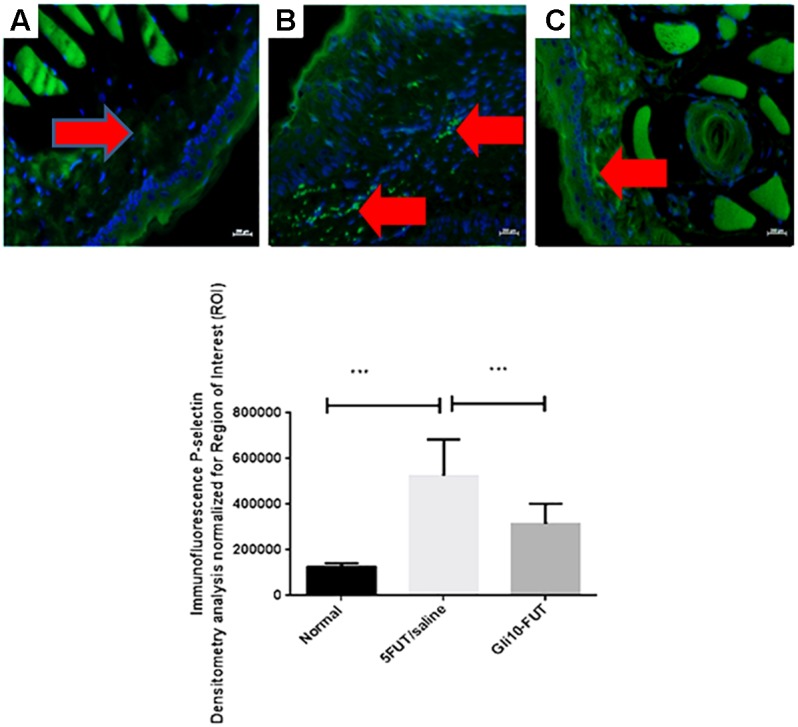
Representative confocal photomicrographs P-selectin immunoreactivity (green) in hamster cheek pouch specimens from each group with DAPI nuclear counterstained (blue) (*n* = 5/group). Normal control hamster cheek pouches presents weak P-selectin **(A)** labeling. Strong P-selectin **(B)** labeling were seen in the 5-FUT/saline group. P-selectin **(C)** labeling was weak in the GLI 10-FUT group. Densitometric analysis confirmed significant decreases in P-selectin immunoreactivity in the normal group compared with 5-FUT/saline group (^∗∗∗^*p* < 0.001). Densitometric analysis confirmed significant decrease in P-selectin immunoreactivity in the GLI 10-FUT group. Five immunofluorescence sections from each animal in each group were analyzed (^∗∗∗^*p* < 0.001, Kruskal–Wallis test followed by Dunn’s test). Scale bar = 50 μm, 40×.

Immunohistochemical analysis was performed in the oral mucosa area, where immunostaining was detected in the cytoplasm and nucleus of epithelial, inflammatory, endothelial and fibroblast cells. The normal group exhibited a negative reaction for COX-2 (*p* < 0.001), iNOS (*p* < 0.001), NFκB P65 (*p* < 0.05), and MMP-2 (p < 0.001) compared to the 5-FUT/saline group. In the normal group, GPx immunostaining was intense when compared to the 5-FUT/saline group (*p* < 0.001; Figure 5). GLI 10-FUT group showed negative immunoreaction for NFκB P65 (*p* < 0.05) and MMP-2 (*p* < 0.001) when compared to the 5-FUT/saline group. With respect to the GLI 10-FUT group, COX-2 and iNOS immunoexpression was weak when compared to the 5-FUT/saline group (*p* < 0.05), however, it appears that for Gpx1, the staining is being restored in the GLI 10-FUT group compared with 5FUT/saline group (*p* < 0.05). Regarding MMP-2, no immunostaining was observed when compared to the 5-FUT/saline group (*p* < 0.001). P-selectin labeling (green) was weak in the normal and GLI 10-FUT groups, and strong in the 5-FUT/saline group (Figure 6). GLI 10-FUT group stimulated the resolution of low inflammation, showing weak P-selectin staining. Densitometric analysis confirmed that there was a significant decrease P-selectin immunoreactivity in the GLI 10-FUT group, compared to the 5-FUT/saline group (*p* < 0.001; Figure 6).

## Discussion

Reactive oxygen species (ROS) produced by 5-FU are thought to account for many of the toxic effects, such as MDA production (Bomfin et al., 2017). The body’s defense against oxidative stress primarily depends on a synergism between enzymes such as glutathione peroxidases (GPx) and non-enzymatic antioxidants (Birben et al., 2012). In the 5-FUT/saline group, a significant increase in lipid peroxidation with MDA formation and absence of GPx immunolabeling was observed in the oral mucosa, and even though the GLI 10-FUT group underwent chemotherapy with 5-FU, a reduction of lipid peroxidation (MDA) with presence of low immunolabeling was observed for GPx.

The cytotoxic effect of H_2_O_2_ is well known, including induction of apoptosis in human normal and cancer cells (Hegedus et al., 2018). There is evidence of gliclazide reducing the number of H_2_O_2_-related necrotic and apoptotic cells, and this effect is most likely caused by free radical scavenging properties (Sliwinska et al., 2012). These properties are related to the presence of a single aminoazabicyclooctane ring in the sulfonylureas, which is considered as capable of inhibiting photo-oxidation (Noda et al., 2000).

5-FU chemotherapy, which acts by the irreversible inhibition of thymidylate synthetase (TS), leads to deoxythymidine and monophosphate shortage (dTNP), damaging the DNA synthesis specifically in the S phase of the cell cycle, mainly acting on proliferative cells (Longley et al., 2003; Can et al., 2013). The animals of the 5-FUT/salive group developed OM with clinical features such as severe erythema, extensive hyperemia, hemorrhagic areas, ulcers and abscesses. Histopathological characteristics in this group showed vascular engorgement and marked vasodilatation; intense cellular infiltration, predominantly polymorphonuclear; presence of hemorrhagic areas, edema, abscesses and extensive ulcers. In our findings, the 5-FUT/saline group had elevated levels of IL-1β and TNF-α.

The GLI 10-FUT group showed a reduction of the anti-inflammatory activity demonstrated by the clinical findings and by the presence of erythema without evidence of mucosal erosion; as well as by histopathological findings with reepithelialization, discrete mononuclear inflammatory infiltration, absence of hemorrhage, edema, ulcers and abscesses. In relation to the inflammatory cytokines, a significant decrease in TNF-α levels was observed in the normal and GLI 10-FUT groups when compared to the 5-FUT/saline group. For IL-1β, a significant reduction was observed in the normal, MT and GLI 10-FUT groups when compared to the 5-FUT/saline group.

Another important marker of the inflammatory process is the cyclooxygenase-2 (Cox-2), which has direct participation in the formation of the prostaglandins involved in the inflammatory process (Gariepy et al., 2017; Mao et al., 2018). Inhibition of Cox-2 is recognized in the success of therapeutic interventions for OM induced by chemotherapy (Sommer et al., 2017; Mahendran et al., 2018). Our findings revealed low Cox-2 immunostaining in the group treated with GLI 10-FUT, corroborating literature data showing the effect of gliclazide in reducing prostaglandins formation (Fujitani et al., 1983; Mizuno et al., 1989).

Excessive production of nitric oxide (NO) in inflammatory processes alters microvascular permeability and plays a pro-inflammatory role (Hatakeyama et al., 2006; Sanchez et al., 2008; Zhou and He, 2011). 5-FU can induce the expression of inducible nitric oxide synthase (iNOS) (Yin et al., 2007). Studies indicate that iNOS reduction by the therapeutic approach or the overexpression of caveolin-1 increases endothelial cell adhesiveness and the interaction of endothelial cell with leukocytes (Fernandez-Hernando et al., 2009, 2010; Atochin and Huang, 2010). In line with these evidences, the present study showed a mild iNOS immunolabeling in the GLI 10-FUT-treated group.

Endothelial adhesion molecules, including E-selectin, P-selectin, ICAM-1 and vascular adhesion molecule 1 (VCAM-1), and leukocyte extravasation during inflammation are particularly regulated by pro-inflammatory NF-κB pathway, which controls transcription of adhesion molecules and mediators of inflammatory processes, such as IL-1β interleukins (Itoh et al., 2003; Lai et al., 2016; Zhao et al., 2017).

Adhesion of inflammatory cells to endothelial cells is linked to the process of radiation-induced damage. As radiation therapy continues, a steady state between mucosal cell death and regeneration may occur due to an increased cell production rate from the surviving cells, in a 05-stages process: (1) Initiation of tissue injury: radiation and/or chemotherapy induce cellular damage causing the death of the basal epithelial cells. The generation of ROS (free radicals) by radiation or chemotherapy is also believed to exert a role in the initiation of mucosal injury. These small highly reactive molecules are by-products of oxygen metabolism and can cause significant cellular damage. (2) Up-regulation of inflammation via generation of messenger signals: in addition to causing direct cell death, free radicals activate second messengers that transmit signals from receptors on the cellular surface to the inside of cell. This leads to up-regulation of pro-inflammatory cytokines, tissue injury, and cell death. (3) Signaling and amplification: up-regulation of pro-inflammatory cytokines, such as TNF-α, produced mainly by macrophages, causes injury to mucosal cells, and also activates molecular pathways that amplify mucosal injury. (4) Ulceration and inflammation: there is a significant inflammatory cell infiltration associated with the mucosal ulcerations, based in part on metabolic by-products of the colonizing oral microflora. Production of pro-inflammatory cytokines is also further up-regulated as a result of this secondary infection. (5) Healing: this phase is characterized by epithelial proliferation, as well as, cellular and tissue differentiation, restoring the integrity of the epithelium (Ps et al., 2009).

Recently, changes in the subepithelial ECM has been recognized as a hallmark of mucositis development. ECM is a complex structural network containing fibrous proteins, proteoglycans and glycoproteins. It provides structural support for the tissue and regulate epithelial cell kinetics. Dysregulation of ECM components, including collagen and fibronectin, has been demonstrated to closely correlate with the observation of maximal tissue damage in mucositis. The cellular receptors that bind to ECMs are referred to as CAMs and include integrins, cadherins and selectins. CAMs are well known for their role in regulation of cell kinetics through ECM regulation. Furthermore, they play a central role in inflammation, which is a key process in the pathogenesis of mucositis (Al-Dasooqi et al., 2017).

P-selectin belongs to the selectin family of leukocyte adhesion receptors and is a transmembrane protein stored in the α-granules of platelets and in the Weibel-Palade bodies of endothelial cells (Barkalow et al., 2000). Chemotherapy-induced mucositis is characterized by damage to mucous membranes. CAMs are have a prominent role in maintenance of normal tissue morphology and wound healing. Specifically, CAMs interact with components of the ECM to regulate cell behavior including apoptosis, proliferation, migration and differentiation. Differential expression of CAMs E-cadherin, P-selectin, E-selectin, and integrin α1 has been demonstrated at the injury and healing phases of mucositis (Al-Dasooqi et al., 2017). In addition, it has been shown that gliclazide inhibits the neutrophils–endothelial cells adhesion and the surface expression of endothelial adhesion molecules (E-selectin, P-selectin, ICAM-1) (Itoh et al., 2003).

Gliclazide inhibits the adhesion of monocytes to endothelial cells, and in this way reduces the glycated albumin levels contributing to the decrease of NFκB activation, resulting in reduced transcription factors of the inflammatory process (Jennings, 2000; Renier et al., 2003). NFκB signaling pathways can be divided into canonical and non-canonical pathways. Canonical NFκB pathway is activated after degradation of IκBα, which results in nuclear translocation of various NFκB complexes, predominantly the p50/p65 dimer. NFκB controls the expression of a number of genes that regulate immune responses, cell growth, proliferation, survival and apoptosis, stress responses and inflammation (Park and Hong, 2016). Our study found a reduction in the P-selectin levels in the GLI 10-FUT group, as well as an absence of the NFκB p65 in the immunoblotting. In turn, the protein quantification of NFκB NLS p50 was reduced when compared to the 5-FUT/saline group.

Several endothelial surface CAMs allow an initial selective adhesion of neutrophils to the tissue damage associated with infiltration of immunoinflammatory cells from the blood and/or lymphatic circulation (Desfaits et al., 1999; Alvaro-Gonzalez et al., 2002). We found that the 5-FUT/saline group showed a significant increase in MPO enzymatic activity, a quantitative marker of neutrophil infiltration in the mucosal inflammation. The GLI 10-FUT group showed a significant reduction of MPO activity, confirming data from the literature that suggests a reduction in the migration of neutrophils and adhesion molecules, such as P-selectin, after gliclazide treatment (Itoh et al., 2003). Our study corroborates these data, since the GLI 10-FUT group also showed reduced P-selectin levels. The selectins are molecules that mediate adhesive interactions between leukocytes, endothelium and platelets. Moreover, the expression of P-selectin have been demonstrated *in vivo* in models of inflammatory tissue (Lorant et al., 1993).

Increase in secretion of ECM degrading enzymes, especially MMP-2 in tissue destruction of inflamed tissues in the jugal mucosa, is evidenced in the high immunolabeling of MMP-2 of the 5-FUT/saline group when compared to the absence of this protein in the GLI 10-FUT, and similar to the saline group. These results are directly due to the pathophysiology of the MMP-2 gelatinase enzyme that degrades denatured extracellular collagen of the type IV extracellular membrane in the presence of inflamed tissues and tissue destruction, differing from the physiological conditions of normal tissue that presents strict regulation of extra MMP secretion by the cells (Kerkela and Saarialho-Kere, 2003; Bodnar et al., 2015).

## Conclusion

**FIGURE 7:**
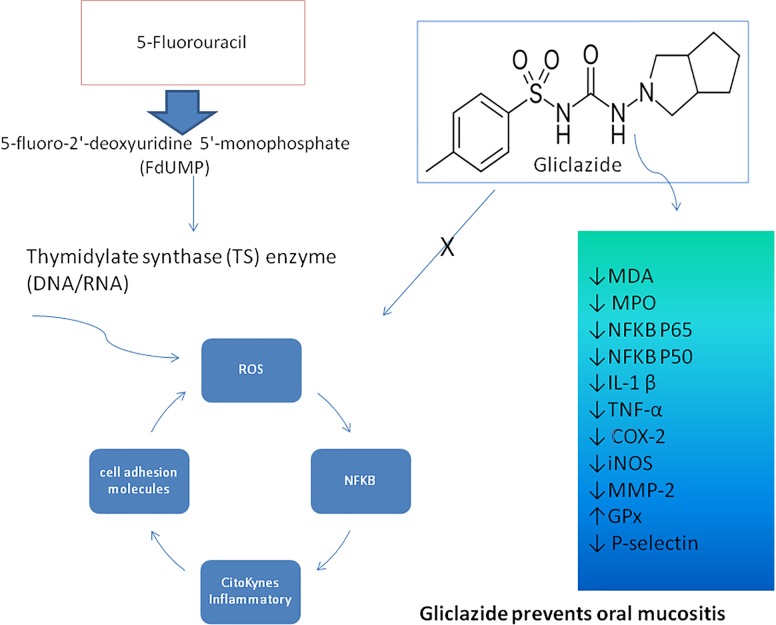
Pharmacological modulation of the 5-FU-induced oral mucositis by Giclazide. DNA/RNA damage due to 5-fluoro-2′-deoxyuridine 5′-monophosphate (FdUMP) activates ROS, NFκB inducing the proinflammatory cytokines, such as IL-1β and TNF-α and cellular adhesion molecules, which promote inflammation and tissue damage in the oral mucosa. Gliclazide interferes with MDA, MPO, NFκB P50, NFκB P65, IL-1β, TNF-α, COX2, iNOS, MMP-2, GPx, and P-selectin, improving oral mucositis.

The results indicate that gliclazide has an anti-oxidant and anti-inflammatory effect, including leukocyte migration and influence on MDA, MPO, NFκB, IL-1β, TNF-α, COX2, MMP-2, iNOS, and P-selectin levels, improving OM (Figure 7). The association between Gliclazide and 5-FU can attenuate the deleterious effects of chemotherapy on OM.

## Author Contributions

CAM, CXM, and AA devised the study design. CAM, CXM, RL, DC, RV, GB, GG, RA, AM, and AA participated in the interpretation of data, and drafted the manuscript. CAM, CXM, RL, DC, RV, GB, GG, RA, AM, and AA carried out the data collection, participated in the interpretation of data, and assisted in the writing the manuscript. CAM, CXM, RL, RV, GG, AM, and AA participated in the interpretation of data and drafted the manuscript. CAM and AA and performed all statistical analysis, participated in the interpretation of data, and assisted in the writing of the manuscript. All authors read and approved the final manuscript.

## Conflict of Interest Statement

The authors declare that the research was conducted in the absence of any commercial or financial relationships that could be construed as a potential conflict of interest.
